# Impact of Nurse-Led Titration Versus Physician Prescription of Hypoglycaemic Agents on HbA1c Level in Type 2 Diabetes Patients: A Systematic Review and Meta-Analysis of Randomized Controlled Trials

**DOI:** 10.7759/cureus.20436

**Published:** 2021-12-15

**Authors:** Suresh K Sharma, Kalpana Thakur, Ravi Kant, Shiv K Mudgal

**Affiliations:** 1 Medical and Surgical Department, All India Institute of Medical Sciences, Jodhpur, IND; 2 Psychiatry and Mental Health Department, All India Institute of Medical Sciences, Rishikesh, IND; 3 Diabetes and Metabolism Division, All India Institute of Medical Sciences, Rishikesh, IND; 4 Medical and Surgical Department, All India Institute of Medical Sciences, Deoghar, IND

**Keywords:** glycated hemoglobin, patients’ satisfaction, dosage titration, diabetic clinic, nurse-led clinic

## Abstract

A nurse-led diabetic clinic to manage type 2 diabetes, which emphasizes medication adherence, titration of hypoglycemic agents, behavior modification, and motivation for lifestyle changes, is widely recommended and practiced in western countries.

This review aims to examine the impact of a nurse-led diabetic clinic versus a standard physician-led diabetic clinic on glycaemic control of type 2 diabetes patients.

Studies were obtained using a comprehensive search in the electronic databases of PubMed, Embase, SCOPUS, Cochrane Library, and gray literature through March 2021. We calculated the pooled effect estimate with 95% confidence intervals (CIs) comparing subjects with and without nurse-led titration of hypoglycemic agents using standardized mean difference (SMD) for continuous outcomes and risk ratio (RR) for dichotomous outcomes.

Four trials comprising 470 participants (241 intervention group and 229 control group) met the inclusion criteria. Glycated hemoglobin (HbA1c levels and BMI were lower in participants with a nurse-led diabetic clinic (SMD = -0.54, 95% CI -0.89 to -0.20, I2 = 67%, p = 0.002) and (SMD = -0.26 (95% CI -0.45 to -0.07, I2 = 0%, p = .008), respectively, than in those attending a standard physician-led diabetic clinic. Similarly, the pooled result shown that patients attending the nurse-led diabetic clinic had a 31% higher satisfaction level (RR 0.69; 95% CI, 0.51 to 0.92, I2 = 0%, p= 0.01). On the other hand, there was no significant association of the nurse-led diabetic clinics on patients’ blood pressure and intensification of hypoglycemic agents. The certainty of the evidence assessed using Grading of Recommendations Assessment, Development, and Evaluation (GRADE) was moderate for outcomes like HbA1c level, intensification of hypoglycemic agents, and patients' satisfaction and low for other secondary outcomes.

Our meta-analysis allows for the conclusion that nurse-led titration of hypoglycemic agents is associated with better glycemic control and enhances patients' satisfaction. Therefore, it is recommended to establish and strengthen nurses-led diabetic clinics for better HbA1c control where physician-led diabetic services are limited. Further research is needed to enhance the quality of the evidence.

## Introduction and background

Diabetes mellitus (DM) is a chronic metabolic illness with a rising prevalence and increased mortality rate. Type 2 diabetes mellitus, where body cells could not utilize or produce insulin, is considered the most typical type as around 90% of the globally identified diabetic population. [1] Around 3.4 million globally and 1 million in South-East Asia die due to diabetes as reported in the 2012 WHO Diabetes fact sheet [2]. As per global trends available from 2019, around 1.5 million deaths were directly linked with diabetes [3]. According to the International Diabetes Federation, 'India is the second-largest hub for diabetes with 77 million patients with diabetes, after China; this number is further expected to reach till 134 million by 2045' [4].

Diabetes increases the risk of severe health issues, and over time, it may damage blood vessels, eyes, kidneys, heart, and nerves. The economic cost of living with diabetes poses a significant challenge to developing countries like India, where 5% to 25% of individuals' earning is spent on diabetes treatment expenditure [5-6] Overall, diabetes has a debilitating impact on the country's economy and health status. Furthermore, the shortage of endocrine specialists in our country (only 650 specialists over a 77 million diabetic population) is another hurdle in diabetes prevention and management [7]. With the scarcity of specialists in the field, it is hard to stop the rising number of diabetic cases-associated complications. Moreover, the involvement of nurses as core team members in the treatment of diabetes could be the best possible solution to address the issues of such a massive diabetic population.

WHO has recently placed more emphasis on an integrated approach and the utilization of other health team members to prevent and manage diabetes-like chronic illnesses [8]. Countries in the West have established nurse-led diabetic clinics while the scenario is the opposite in south-east Asian countries because the medical fraternity is still a little reluctant to support nurse-led diabetic clinics [9]. The establishment of a nurse-led diabetic clinic would be an essential step towards professional advancement and autonomy in nursing as a whole [9-10]. A nurse-led diabetic clinic includes patients’ assessment, education, skill training in insulin administration, titration of dosages for hypoglycemic agents, and regular follow-up. Few studies have evaluated that nursing care in diabetes brings positive outcomes in terms of glycemic control and patients' satisfaction, but there are still contradictory findings [11-14]. However, a recent meta-analysis on the feasibility of nurse-led clinics and nurse-led prescriptions concluded that nurse-led follow-up on dosage titration would be equally effective as physicians' prescriptions [15]. The current review was conducted to examine the impact of the nurse-led diabetic clinic and standard physician-led diabetic clinic on the glycated hemoglobin (HbA1c) level of diabetes mellitus (DM) type 2 patients. This investigation may reveal the potential effectiveness of a nurse-led approach in diabetes management with updated studies in the field. Unlike the previous review, this meta-analysis excludes studies where nurse-led education was an intervention. Furthermore, policymakers and stakeholders in the nursing profession might get updated literature to analyze clinical outcomes or differences created on utilizing a nurse-led treatment modality, especially in chronic illnesses. 

## Review

Methods

Review Question

What is the impact of a nurse-led diabetic clinic versus a standard physician-led diabetic clinic on glycaemic control in type 2 diabetes patients? This investigation may reveal the potential effectiveness of intervention in diabetes management.

Protocol and Registration

The systematic review was conducted on the available literature related to the topic or review question. Preferred Reporting Items for Systematic Reviews and Meta-Analyses (PRISMA) was used for systematically carrying out the current review (http://www.prisma-statement.org) [16]. The protocol for this systematic review and meta-analysis was registered on the International Prospective Register of Systematic Reviews (PROSPERO), entitled “Impact of nurse-led titration versus physician prescription of hypoglycaemic agents on HbA1c level in type 2 diabetes patients: A systematic review and meta-analysis of randomized controlled trials,” reference number CRD42020172576. The registered protocol is available at: https://www.crd.york.ac.uk/prospero/display_record.php?ID=CRD42020172576.

Inclusion Criteria

Population, Interventions, Comparisons, and Outcomes (PICO) was considered for building initial eligibility and search criteria. Only randomized controlled trials of patients older than 18 years of age and diagnosed with diabetes type 2 were included in this review. Intervention for this review and meta-analysis was drug dosage titration follow-up by a registered nursing professional in an outpatient department. The intervention group was compared against standard treatment in a diabetic clinic by a physician. Primary outcomes included glycaemic control (HbA1c level) and secondary outcomes were body mass index (BMI), blood pressure, intensification of hypoglycemic agents, patient satisfaction, hypoglycemic adverse events, and patient's quality of life (Table 1).

**Table 1:** Nurse-Led Titration compared to Physician's Prescription for HbA1c Level in Type 2 Diabetes Patients

Patient or Population: HbA1c Level in Type 2 Diabetes Patients; Intervention: Nurse-Led Titration; Comparison: Physician's Prescription					
Outcomes	No. of participants (studies) follow-up	Certainty of the evidence (GRADE)	Relative effect (95% CI)	Anticipated absolute effects	
				Risk with physician's prescription	Risk difference with nurse-led titration
HbA1c level assessed with: Laboratory values of glycated haemoglobin scale from 4% to 13% follow-up: range 6 months to 14 months	470 (4 RCTs)	⨁⨁⨁◯ MODERATE ^a^	-	The mean hbA1c level ranged from 7.3-8.9 %	MD 0.54 % lower (0.89 lower to 0.2 lower)
BMI assessed with: Weight (kg)/Height (meters squared); Scale from: 18.5 to 30 follow-up: range 6 months to 14 months	424 (3 RCTs)	⨁⨁◯◯ LOW ^a,b^	-	The mean BMI ranged from 26.2-30.3	MD 0.07 lower (0.5 lower to 0.35 higher)
Blood Pressure assessed with: Sphygmomanometer/Automatic Blood Pressure Device follow-up: range 6 months to 14 months	339 (3 RCTs)	⨁⨁◯◯ LOW ^a,c^	RR 0.90 (0.98 to 1.07)	Low	
				10 per 100 ^d^	1 fewer per 100 (0 fewer to 1 more)
				High	
				25 per 100 ^d^	2 fewer per 100 (1 fewer to 2 more)
Intensification of hypoglycaemic agents assessed with: medication adjustment record follow0up: range 6 months to 14 months	250 (3 RCTs)	⨁⨁⨁◯ MODERATE ^e^	RR 0.31 (0.74 to 1.74)	Low	
				50 per 100 ^f^	35 fewer per 100 (13 fewer to 37 more)
				High	
				60 per 100 ^f^	41 fewer per 100 (16 fewer to 44 more)
Patients' satisfaction assessed with: patients' satisfaction response question follow-up: range 6 months to 14 months	424 (3 RCTs)	⨁⨁⨁◯ MODERATE ^a^	RR 0.52 (0.80 to 1.22)	Low	
				10 per 100 ^g^	5 fewer per 100 (2 fewer to 2 more)
				High	
				60 per 100 ^g^	29 fewer per 100 (12 fewer to 13 more)
*The risk in the intervention group (and its 95% confidence interval) is based on the assumed risk in the comparison group and the relative effect of the intervention (and its 95% CI). CI: Confidence interval; MD: Mean difference; RR: Risk ratio. Explanations a. No allocation concealment in one study. b. One study reported incomplete baseline values. c. One study weighted heavily in the meta-analysis and that could affect the consistency of results. d. The low and high-risk values are the two extreme numbers of control group study participants with a change in blood pressure from the studies included in the review. e. One study did not mention the direct details of medication intensification and that could affect directness. f. The low and high-risk values are the two extreme numbers of control group study participants with a change in the intensification of hypoglycemic agents from the studies included in the review. g. The low and high-risk values are the two extreme numbers of control group study participants with a change in patients' satisfaction from the studies included in the review.					
GRADE Working Group grades of evidence: High certainty: We are very confident that the true effect lies close to that of the estimate of the effect. Moderate certainty: We are moderately confident in the effect estimate: The true effect is likely to be close to the estimate of the effect, but there is a possibility that it is substantially different. Low certainty: Our confidence in the effect estimate is limited: The true effect may be substantially different from the estimate of the effect. Very low certainty: We have very little confidence in the effect estimate: The true effect is likely to be substantially different from the estimate of effect.					

Data Sources and Risk Bias Assessment

A search strategy was developed to retrieve all relevant information on nurse-led titration in the treatment of patients with diabetes. The search was initially conducted till March 2021 and later updated in July 2021. The investigation aimed to find published studies in the English language, and there was no date restriction while searching. The complete search was undergone in a three-step process. For the initial step, a senior librarian was consulted to get assistance in the initial searching of the PubMed, EMBASE, SCOPUS, and Cochrane Library databases, with optimal search terms. An extensive advance search was done in the second step using all identified keywords, index terms, and boolean operators for each database (Appendices: Search Strategy). The last step was to search reference lists of the relevant articles.

Initially, two independent reviewers screened studies' titles and abstracts for full-text eligibility, and the discrepancy was resolved by discussing the two. Further, the Cochrane risk of bias tool was used for methodological validity of the included studies for domains such as randomization process, allocation concealment, blinding of participants and outcome assessor, and incomplete and selective outcome reporting [17]. Queries were raised to the corresponding authors of all included studies wherever required. All the authors reviewed included studies for methodological quality, and discrepancies were resolved by mutual consensus. A risk of bias graph and a risk of bias summary were prepared on the extracted information.

Data Synthesis and Analysis

Data synthesis was based on baseline and post-intervention data of both the groups (intervention and comparator) to evaluate intervention effectiveness based on reported outcomes in included studies. Review Manager (Version 5.3) was used for carrying out a meta-analysis of included RCTs [18]. The approach for meta-analysis was determined by the type of reported and interpreted outcome data. For continuous data (HbA1c and BMI), the standardized mean difference (SMD) associated with 95% confidence intervals were considered, and dichotomous data were analyzed using relative risk associated with 95% confidence intervals across all included RCTs. A random-effect model instead of a fixed model was preferred for this meta-analysis to minimize the risk of heterogeneity. To evaluate heterogeneity, the I2 statistic was used, and >50% was considered as significant heterogeneity. A narrative synthesis was framed for the findings or outcome where data could not be pooled for meta-analysis.

The Grading of Recommendations Assessment, Development, and Evaluation (GRADEpro) software was used to create a summary of findings table and critical judgment on the quality of evidence [19]. This quality of the evidence would be helpful in projecting confidence in an estimated effect to support our intervention. The certainty of the evidence assessed using GRADE was moderate for outcomes like HbA1c level, intensification of hypoglycemic agents, and patients' satisfaction and low for other secondary outcomes (Table 1).

Results

Characteristics of the Included Studies

**Figure 1:**
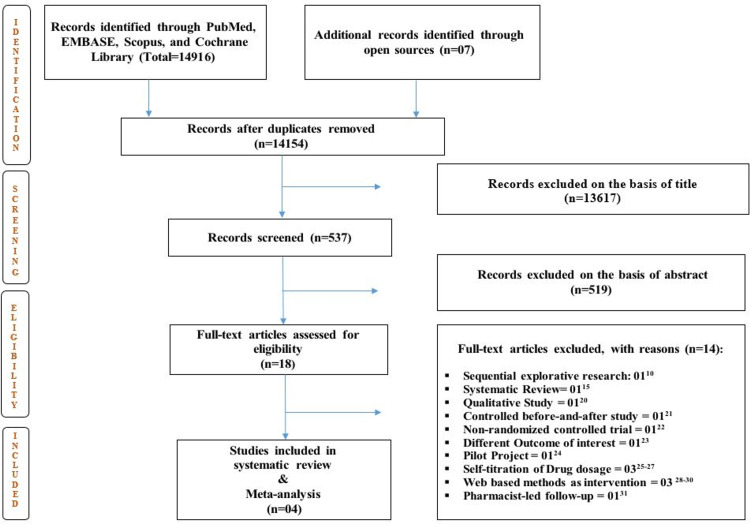
PRISMA flow chart PRISMA: Preferred Reporting Items for Systematic Reviews and Meta-Analyses

A total of 14916 records were identified after a thorough search of databases. Studies were screened for duplicate records and on the basis of the title. Further, 537 studies' abstracts were examined to ensure their relevance for inclusion. Total 519 studies were excluded based on abstract because of variation in the intervention (nurse-led educational/training program was used to manage patients with diabetes). Full-text eligibility was assessed for 18 studies, out of which 14 were excluded, and the rationale for exclusion was mentioned in the PRISMA flow chart (Figure 1) [10,15,20-31]. Finally, four RCTs were included for the purpose of systematic review and meta-analysis [11-14].

Risk of Bias

**Figure 2:**
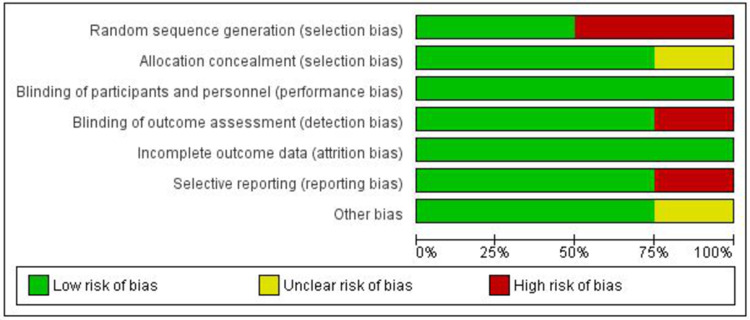
Risk of Bias Graph: Review Authors’ Judgments About Each Risk of Bias Item Presented as Percentages Across All Included Studies

**Figure 3:**
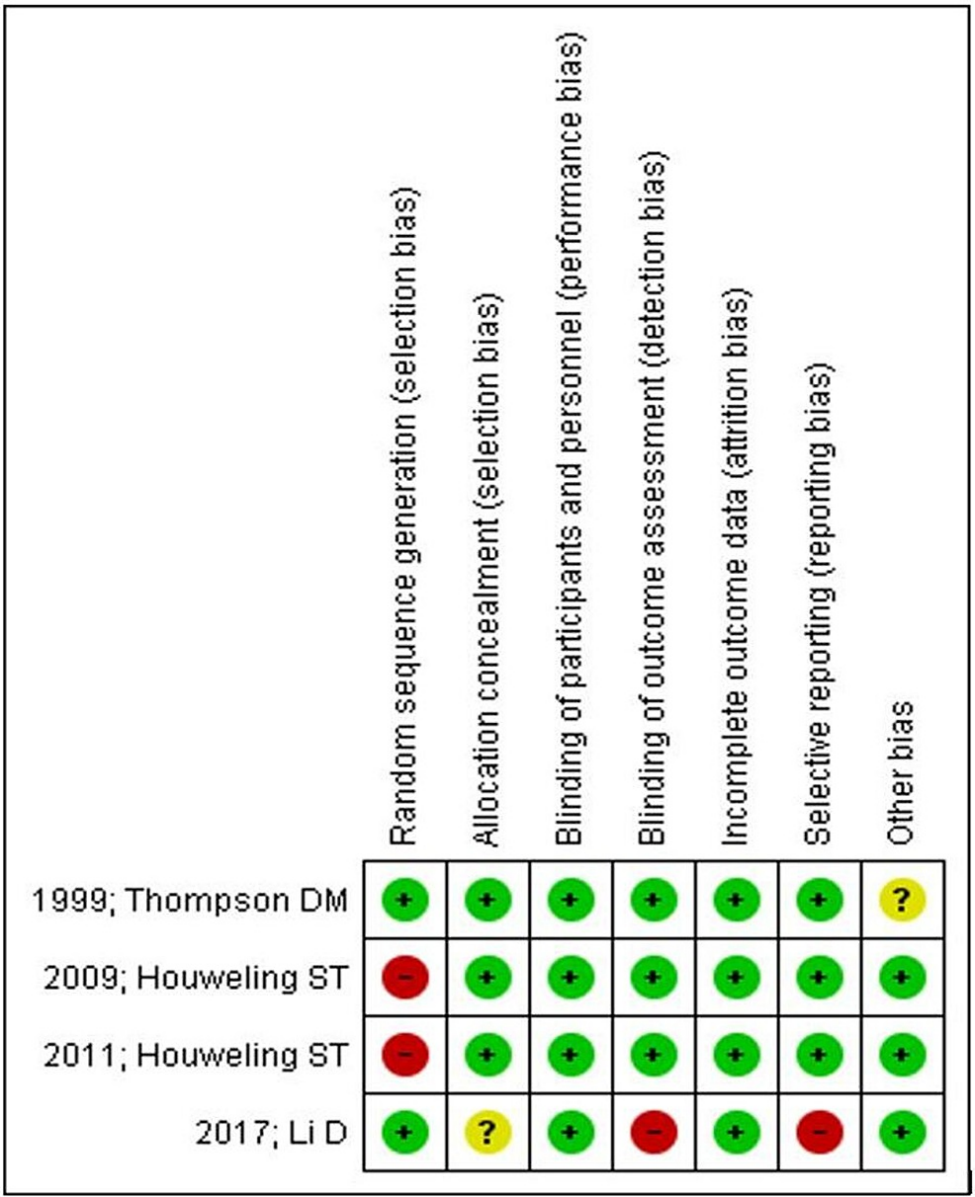
Risk of Bias Summary: Review Authors’ Judgments About Each Risk of Bias Item for Each Included Study

Two [12-13] of the four included studies [11-14] were identified as high risk of bias for the process of randomization because it was performed on the basis of odd and even numbers (even to intervention and odd to control). Although there was one study that mentioned the randomization process, it could not explain the method used for allocation concealment, envelopes used were sealed and sequentially numbered, but it lacks clarity on whether it was identical, opaque, or transparent [14]. All the included studies were at low risk of bias, as blinding of participants and personnel was performed in all included RCTs. Incomplete reporting for the study's outcome, i.e., BMI as the baseline values, was not mentioned for both the groups in a study carried out by Li D et al. [14]. Furthermore, the same study was at high risk for outcome assessment blinding because the patient-selected laboratory was preferred. Still, in some cases, a nurse performed point of care testing, which could influence overall outcome measurement. One study appears to be unclear for outcome reporting, for example, hypoglycemic events were not mentioned with clarity (Figures 2-3) [11]. More than 400 participants, i.e., 470, from all included studies, and therefore, chances of imprecision are less. Furthermore, all the included studies have uniformity in terms of reported intervention (nurse-led titration) and primary outcome (HbA1c levels); hence, an appropriate conclusion can be drawn from study results (Table 2).

**Table 2:** Risk of Bias Appraisal for the Included Studies

Study	Randomization	Allocation	Participant & Personnel Blinding	Outcome Blinding	Incomplete Outcome Data	Selective Reporting	Other Bias
Thompson DM [11], 1999	Low Risk- Patients were randomized using random number tables	Low Risk- Sequentially numbered opaque sealed envelopes were used for allocation concealment	Low Risk- Participants were not blinded but as per authors’ judgment, it would not impact study outcome	Low Risk- Laboratory technicians performing the outcome assessment had no knowledge about the study and were blinded to the patients’ assignment group	Low Risk- The study has reported all the outcomes as per the pre-specified objectives and there was no reported attrition	Low Risk- The study’s primary & secondary outcomes were reported as per specified objectives	Unclear Risk- The study appears to be unclear for a few outcomes such as hypoglycaemic events were not mentioned with clarity
Houweling ST [12], 2009	High Risk- Randomized clients on the basis of odd and even (Even to intervention and odd to control)	Low Risk- Non-transparent, closed, sequentially numbered envelopes were used for allocation concealment	Low Risk- Participants were not blinded but as per authors’ judgment, it would not impact study outcome	Low Risk- Independent medical investigator performed outcome assessment prior to the study and after 6 months and 12 months	Low Risk- The reason for participants’ attrition was mentioned and missing information is unlikely to be related to the true outcome	Low Risk- Study’s (primary & secondary) outcomes were reported as per specified objectives	Low Risk- The study appears to be free from other bias
Houweling ST [13], 2011	High Risk- Randomized clients on the basis of odd and even (even to intervention and odd to control)	Low Risk- Non- transparent, closed, sequentially numbered envelopes were used for allocation concealment	Low Risk- Participants were not blinded but as per the authors’ judgment, it would not impact the study outcome	Low Risk- Outcome assessment was performed anonymously by two independent medical investigators after 6 months and 12 months	Low Risk- The reason for the participants’ attrition was mentioned and missing information is unlikely to be related to the true outcome	Low Risk- The study’s (primary & secondary) outcomes were reported as per specified objectives	Low Risk- The study appears to be free from other bias
Li D [14], 2017	Low Risk- Randomized clients using stratified permuted block randomization	Unclear Risk- Envelopes used were sealed and sequentially numbered but it lacks clarity whether it was identical, opaque, or transparent	Low Risk- Participants were not blinded but as per the authors’ judgment, it would not impact the study outcome	High Risk- A patient-selected laboratory was preferred but in some cases, NCM performed point-of-care testing, which could influence outcome measurement	Low Risk- The reason for participants’ attrition was mentioned and missing information is unlikely to be related to true outcome. Lost to follow-up was not different in the 2 groups	High Risk- One of the study’s outcomes, i.e. BMI, was reported incompletely, as the baseline values were not mentioned for both the groups	Low Risk- The study appears to be free from other bias

Glycemic Control (HbA1c Level)

**Figure 4:**
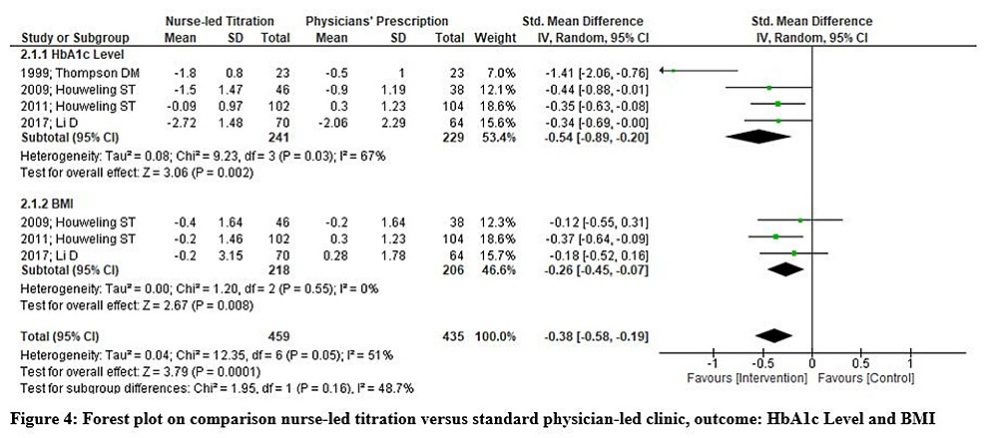
Forest Plot on Comparison of Nurse-Led Titration Versus Standard Physician-Led Clinic; Outcome: HbA1c Level and BMI HbA1c: glycated hemoglobin; BMI: body mass index

**Figure 5:**
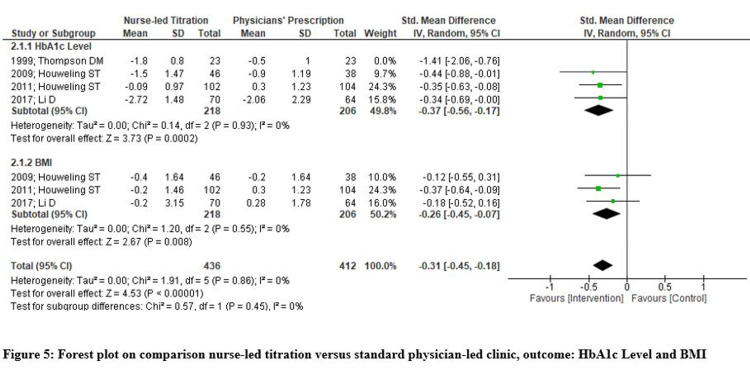
Forest plot on comparison nurse-led titration versus standard physician-led clinic, outcome: HbA1c Level and BMI HbA1c: glycated hemoglobin; BMI: body mass index

Four studies reported HbA1c levels for all the study participants, and baseline parameters indicated that HbA1c levels were high for participants in both groups. The HbA1c level significantly reduced in the nurse-led titration group with a standardized mean difference of -0.54 (95% CI -0.86, -0.20; p=.002; I2= 67%). A random-effect model was used, but significant heterogeneity was still reported, with I2=67%. Due to the limited number of RCTs, a sub-group analysis could not be done, and therefore, authors excluded one study performed on a few less samples and found that there was a statistically significant reduction in HbA1c levels of patients receiving nurse-led titration with a standardized mean difference of -0.34 (95% CI -0.69, -0.00; p<.001; I2= 0%). Hence, pooled analysis from studies reported that nurse-led titration of hypoglycemic agents has a positive impact on patients' glycemic control (Figure 4-5).

Body Mass Index (BMI)

There were three RCTs that reported participants' BMI changes and found a statistically significant difference favoring nurse-led titration with a standardized mean difference of -0.26 (95% CI -0.45, -0.07; p=.008; I2= 0%). Hence, pooled analysis with no heterogeneity indicates that nurse-led titration of hypoglycemic agents effectively achieved a positive impact on participants’ body mass index (Figures 4-5).

Blood Pressure (BP)

**Figure 6:**
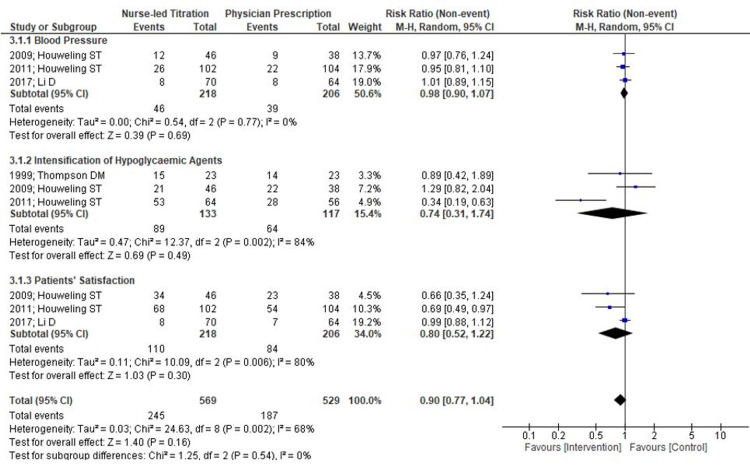
Forest Plot on Comparison Nurse-Led Titration Versus Standard Physician-Led Clinic; Outcome: Blood Pressure, Intensification of Hypoglycemic Agents, and Patients’ Satisfaction

**Figure 7:**
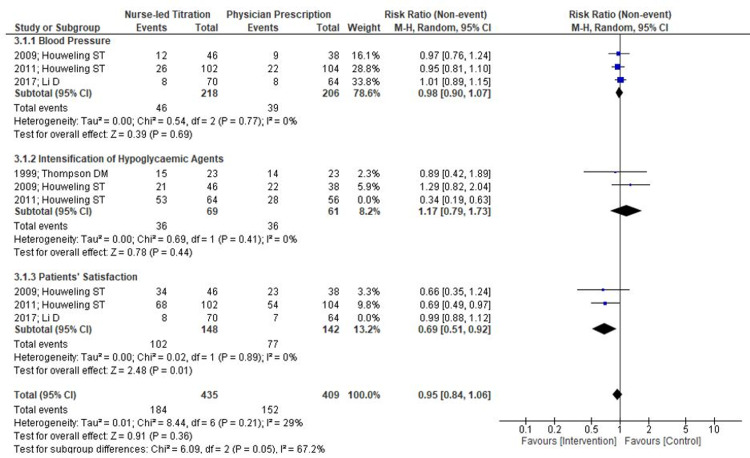
Forest Plot on Comparison Nurse-Led Titration Versus Standard Physician-Led Clinic; Outcome: Blood Pressure, Intensification of Hypoglycemic Agents and Patients’ Satisfaction

Three RCTs measured the incidence rate of blood pressure improvement on a pre-defined goal of > 140/90 mmHg. The number of participants meeting predefined blood pressure goals were more in the nurse-led titration group (46/218) than the physician’s prescription group (39/206) but it was not statistically significant (RR=0.98; 95% CI 0.90, 1.07; p= .77; I2= 0%) (Figures 6-7).

Intensification of Hypoglycemic Agents

Only three randomized controlled trials (RCTs) mentioned an intensification of dosage. Although there are no uniform guidelines on intensifying different hypoglycemic agents, authors have performed a pooled analysis of outcome data based on the number of participants with an intensified dosage of hypoglycemic agents in both groups. There was no statistical significant difference between follow-up by nurse or physician for the intensification of drug dosage in diabetes treatment (RR=0.74; 95% CI 0.31, 1.74; p= .84; I2= 0%) (Figure 6-7).

Patients’ Satisfaction

Satisfaction with treatment was assessed in three studies. Further, meta-analysis of the patients’ satisfaction scores showed no statistical significant difference between nurse-led titration and physicians’ prescription (RR=0.80; 95% CI 0.52, 1.22; p= .30; I2= 80%). Although a random effect model was used for pooled analysis, results still showed severe heterogeneity. Further, authors excluded one study performed in different country and analysis demonstrated statistically significant difference in favor of nurse-led titration versus physicians’ prescription (RR=0.69; 95% CI 0.51, 0.92; p= .01; I2= 0%) (Figures 6-7).

Others

Hypoglycemic events: Hypoglycemic adverse events were monitored only in two studies [11,14]. Li D et al. reported no reported occurrences of hypoglycemia in either group [14]. On the other hand, Thompson DM et al. stated that four hypoglycemic reactions were noted and managed by the nurse in the intervention group, but no reliable documentation was available for the control group participants. [11] Unfortunately, because of discrepancies in outcome reporting in the papers, no meta-analysis could be done. But it was evident that there was clearer documentation of adverse events by patients attending nurse-led follow-up clinics than physicians' clinics.

Quality of life: The impact of nurse-led titration versus physicians' prescription on patients' quality of life was monitored in two studies [13-14]. Different tools, i.e., Patient Health Questionnaire - 9 (PHQ-9) and 36-item Short-Form Health Survey (SF-36), were used to monitor the quality of life, and therefore, a meta-analysis could not be performed for pooled analysis. The results of both studies were contrary because one study [13] concluded that nurse-led clinics help improve patients' quality of life. At the same time, another presented an unexpected result that shows that patients attending nurse-led clinics had deterioration in physical components scores [14].

**Figure 8:**
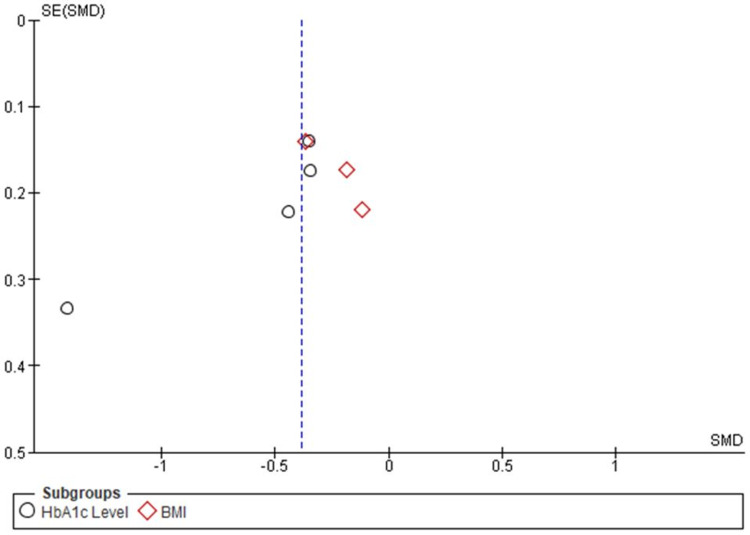
Funnel Plot of Comparison Nurse-Led Titration Versus Standard Physician-Led Clinic; Outcome: HbA1c Level and BMI HbA1c: glycated hemoglobin; BMI: body mass index

**Figure 9:**
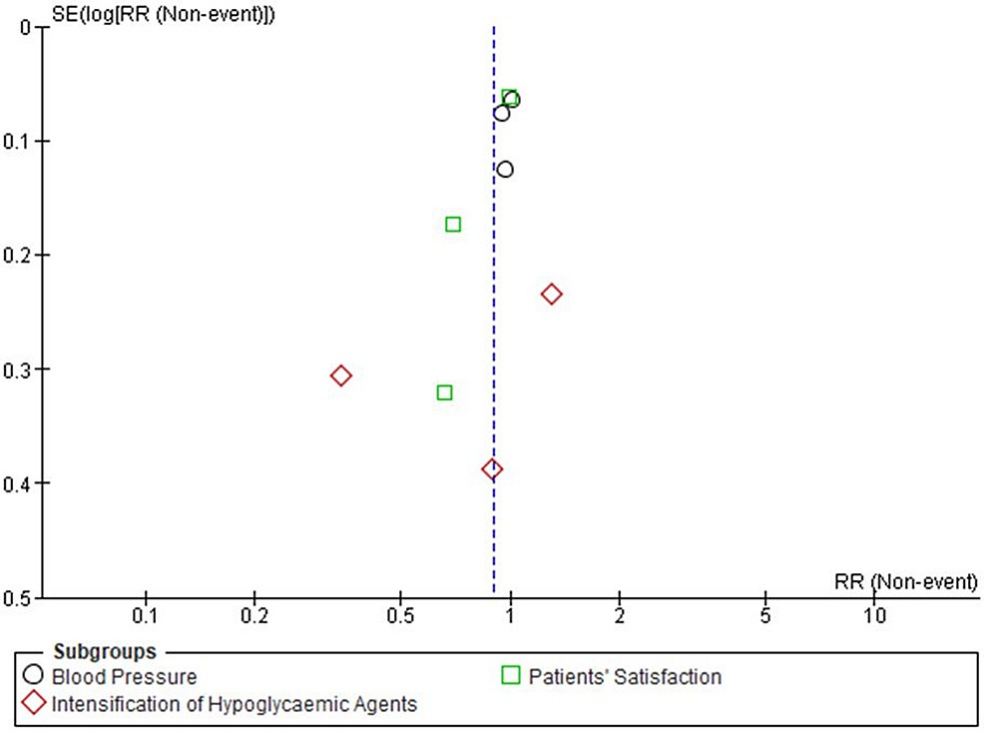
Funnel plot of comparison nurse-led titration versus standard physician-led clinic, outcome: Blood Pressure, Intensification of Hypoglycemic Agents and Patients’ Satisfaction.

Publication bias: A funnel plot was framed to identify the publication bias in the four included trials against all clinical outcomes such as HbA1c level, BMI, blood pressure, intensification of hypoglycemic agents, and patients’ satisfaction. Funnel plot asymmetry indicated the risk of publication bias. Further, Egger’s test could be performed to explore publication bias and understand the effect of estimates of intervention on their standard errors but due to the limited number of studies, authors did not perform this test (Figures 8-9).

Discussion

A total of four RCTs form the basis for this systematic review and meta-analysis. Nurse-led titration of hypoglycemic agents in a routine diabetic clinic was the central focus as an intervention. Patients' education, skill training for self-monitoring of blood glucose levels, and regular follow-up were some of the other activities performed by diabetic nurses. The present meta-analysis was performed for outcomes like HbA1c level, BMI, blood pressure, intensification of hypoglycemic agents, and patients’ satisfaction. The findings of the present study suggest that patients who attended nurse-led clinics reported positive clinical outcomes. Also, the involvement of nurses in the treatment did not cause any harmful effects to diabetic patients. A nurse-led diabetic clinic managed by nurses in collaboration with a physician can be the outsmart approach to deal with the increasing number of diabetic cases and associated complications.

Diabetes can be identified by different diagnostic measures such as fasting plasma glucose, postprandial glucose test, and oral glucose tolerance test. But keeping in mind the inconvenience while using these methods, HbA1c level has been recommended by an international committee and by the American Diabetes Association (ADA) to diagnose diabetes [32]. As per the WHO report, HbA1c testing is also beneficial in the early identification of microvascular complications [33]. Each diabetic patient seeks better glycemic control to avoid the risk of complications and better quality of life. Our meta-analysis suggested a significant reduction in the HbA1c level of patients attending nurse-led diabetic clinics compared to standard physicians' clinics. Our result is in line with other studies stating that the nurse specialist gives care similar to that of the physician in diabetes management [20,22-23]. Strengthening the importance of independence in the treatment of diabetes, one study emphasized that patient-managed dosage titration for simple bolus insulin is as effective as physician-driven dosage adjustment [26]. Long-term blood glucose control was achieved by diabetic patients when provided with pharmacist-driven services [31]. Transformation of care where nursing manpower can be utilized in managing the burden of chronic illnesses is a beginning step towards nurses' autonomy and optimum health outcomes.

Diabetes can be debilitating, and patients always remain on the verge of complications if proper treatment and lifestyle changes are not adopted on time. Studies confirmed that those who are overweight and obese have higher chances of being pre-diabetic and diabetic [34-36]. Patients attending nurse-led diabetic clinics directly communicated with nurses regarding their lifestyle and dietary advice. Therefore, pooled analysis of results in the present meta-analysis shows a significant decrease in BMI of the patients attending nurse-led clinics compared to physicians' clinics. Price C et al. in their study demonstrated that patients with BMI levels less than 27.0 benefitted more from the nurse-led intervention than those with obesity [21]. A study performed on insulin initiation, and titration suggested that individual treatment rather than group treatment effectively controlled body weight [27]. On the contrary, a study on the difference in insulin intensification between patient self-management versus physicians’ management revealed a higher mean weight for the patients managed group [26]. Furthermore, enough literature is available to support the fact that blood pressure control is crucial for managing clinical outcomes in diabetic clients, as it eliminates or minimizes the risk of complications like heart failure, renal disease, and mortality [37-39]. As per our results, there was no significant difference in patients’ blood pressure whether they had attended nurse-led diabetic clinic or physicians’ led standard clinic. One study stated that blood pressure in the patient who received nursing services was decreased by 8 mmHg, but there was no improvement in the standard treatment group [14]. On the contrary, it was explained in one of the study results that the cholesterol/high density lipoprotein (HDL) ratio was less in patients attending a physician-led clinic [12].

Additionally, few studies mentioned the intensification of hypoglycemic agents, but findings lack clarity in terms of dosage titration. Although it can be concluded from our meta-analysis that an equal number of participants in both groups (nurse-led clinic versus physician clinic) received dosage intensification. Each physician prefers different treatment plans for diabetes treatment; therefore, tracing the exact dosage of drug intensification is difficult. ADA has given recommendations on dosage intensification for patients with uncontrolled HbA1c levels [40]. This step is initiated to prevent the risk of complications and to empower patients in self-management. In the same context, if patients can perform recommended dosage titration, nurses who are experts in the field should be given the opportunity to work for patients' treatment. In our review, it was important to note that diabetic nurses recorded hypoglycemic events, but there was no tracing of hypoglycemia in the standard treatment group. A study on automated insulin dosage adjustment showed that hypoglycemia events were more in the intervention group, and frequent dose changes could be the reason [30]. Therefore, it is crucial not only to focus on drug titration but also on patients as a whole while making changes in a treatment plan.

Overall wellbeing is affected by diabetes, and therefore, diabetes distress may be reported by many patients with a chronic history. One study said that patient who was meeting diabetic nurse regularly for treatment had shown a reduction in their distress score from moderate to low while in the standard treatment group, there was no change [14]. Our study findings suggested that more patients were satisfied with the treatment and care in nurse-led clinics than standard care diabetes treatment. Nurse-led models received an overwhelming response from patients, as their unresolved queries are resolved and they felt more confident in dealing with diabetes. Moreover, physicians involved in the study have also accepted that nurses' involvement in diabetes management can create a positive difference in patients' recovery [20]. It was also reported in one of the studies that nurses spent an average of 128 minutes with each patient during the study period. In contrast, physicians only spent 28 minutes with patients, which could also be the reason for patients’ satisfaction in favor of nursing services [13]. Two studies have also highlighted that specialist nurses were more focused on the titration of hypoglycemic agents while physicians were keener on targeting lipid levels. Courtney et al. in their study concluded that incidences of medication errors were less in nurse-led diabetic clinics [22], while another study reported that follow-up by nurses generates similar results to that of physicians’ follow-up in terms of quality and cost-effectiveness [23]. This all indicates that nurses specializing in diabetes management can provide high-quality treatment and follow-up services to the patient, eventually decreasing the diabetes-associated healthcare burden.

## Conclusions

Increased number of people with diabetes and estimated future numbers have exhausted the health care system. Physicians alone cannot manage such a huge diabetic population, and ultimately, quality of care is compromised. The applicability of the nurse-led model in treating chronic illnesses is well-appreciated in the western world, but in south-east Asian countries, it still lacks recognition. Effective utilization of specialized nurses will be helpful in terms of clinical outcomes and a strengthened and equipped health care system. There were no negative results reported for nurse-led titration, and the patients were more satisfied with nursing services. This conveys that nurses can add value to diabetic treatment and management if they are adequately trained and specialized in diabetes to meet the expected quality standards.

According to current review results, nurse-led titration and diabetic clinics are comparable to others and bring better glycemic control and satisfaction. Nurses' authority for prescription always remains a big question and therefore, a joint clinic approach where nurses collaborate with physicians could be best to provide holistic care. Nurses in such clinics are well-versed and authorized to titrate drug dosage, provide referral, and order laboratory investigations. Further RCTs are recommended on nurse-led diabetic clinics, especially from south-east Asian countries to globally establish the effectiveness of this nurse-led model in diabetes care. It is also suggested to conduct a systematic review on the cost-effectiveness of nurse-led diabetic clinics.

## Appendices

Search strategies

**Table 3:** Electronic Database Search Strategy

Pubmed Central	("Diabetes Mellitus, Type 2"[Mesh]) AND "Hypoglycemic Agents"[Mesh] (("Primary Nursing"[Majr]) AND "Blood Glucose"[Mesh]) OR "Blood Glucose Self-Monitoring"[Mesh] ("Diabetes Mellitus, Type 2"[Majr]) AND "Practice Patterns, Nurses'"[Mesh] (("Practice Patterns, Nurses'"[Mesh]) OR "Nurse Clinicians"[Mesh]) OR "Nurse's Role"[Mesh] "Insulin, Ultralente"[Mesh] OR "Insulin, Lente"[Mesh] OR "Biphasic Insulins"[Mesh] ("diabetes mellitus, type 2"[MeSH Terms] OR "Diabetes Complications"[MeSH Terms] OR "Diabetes Mellitus"[MeSH Terms]) AND ("practice patterns, nurses"[MeSH Terms] OR "Nurse Clinicians"[MeSH Terms] OR "Nurse's Role"[MeSH Terms]) "diabetes mellitus, type 2"[MeSH Terms] AND "Hypoglycemic Agents"[MeSH Terms] AND ("practice patterns, nurses"[MeSH Terms] OR "Nurse Clinicians"[MeSH Terms] OR "Nurse's Role"[MeSH Terms]) ("practice patterns, nurses"[MeSH Terms] OR "Nurse Clinicians"[MeSH Terms] OR "Nurse's Role"[MeSH Terms] OR "Physicians"[MeSH Terms]) AND ("Time-to-Treatment"[MeSH Terms] OR "Treatment Outcome"[MeSH Terms]) ("practice patterns, nurses"[MeSH Terms] OR "Nurse's Role"[MeSH Terms] OR "Physicians"[MeSH Terms]) AND "Treatment Outcome"[MeSH Terms]) AND "Diabetes Mellitus, Type 2"[Mesh] ("Glycated Hemoglobin A"[Mesh]) AND "Nurse Clinicians"[Mesh] ("Diabetes Mellitus, Type 2"[Mesh]) AND "Nurse Clinicians"[Mesh]) ("Drug Prescriptions/nursing"[Mesh]) AND ( "Hypoglycemic Agents/administration and dosage"[Mesh] OR "Hypoglycemic Agents/therapeutic use"[Mesh] )
Embase Search Strategy	(('non insulin dependent diabetes mellitus' OR 'antidiabetic agent') AND 'nursing intervention' AND physician AND ('treatment outcome'/exp OR 'treatment outcome') OR 'hemoglobin a1c'/exp OR 'hemoglobin a1c') 'diabetes mellitus' AND 'drug dose titration' AND 'hemoglobin a1c' ('antidiabetic agent' OR 'drug dose titration') AND 'clinical nurse specialist' AND 'glucose blood level' (('antidiabetic agent' OR 'drug dose titration') AND 'clinical nurse specialist' OR 'conservative treatment' OR 'routine outcome monitoring') AND 'hemoglobin a1c' 'diabetes mellitus' AND 'drug dose titration' AND 'glucose blood level' ('diabetes mellitus' OR 'non insulin dependent diabetes mellitus') AND 'clinical nurse specialist' insulin AND 'drug dose titration' AND 'clinical nurse specialist' AND 'hemoglobin a1c' OR 'glucose blood level' (insulin OR 'drug dose titration') AND 'clinical nurse specialist' AND 'clinical practice' AND 'hemoglobin a1c' insulin AND 'drug dose titration' AND 'clinical practice' AND 'hemoglobin a1c' insulin AND 'drug dose titration' AND 'nursing intervention' NOT 'educational model' AND 'hemoglobin a1c' OR 'glucose blood level'
Scopus	high AND pressure AND oxygen AND therapy AND versus AND standard AND treatment hyperbaric AND oxygen AND therapy AND in AND treatment AND of AND diabetic AND foot hyperbaric AND oxygen AND therapy AND versus AND standard AND treatment AND for AND diabetic AND foot AND ulcer AND treatment oxygen AND therapy OR standard AND treatment AND diabetic AND foot OR foot AND ulcer OR chronic AND wound diabetic AND foot OR chronic AND wound AND hyperbaric AND oxygen AND therapy AND versus AND standard AND routine AND treatment
Cochrane Library	("nurse") OR ("nurse's aide") OR ("nurse clinician") AND ("diabetes mellitus") OR ("glycemic control") ("nurse"):ti,ab,kw OR ("nurse's aide"):ti,ab, kw OR ("nurse clinician"):ti,ab,kw AND ("diabetes mellitus"):ti,ab,kw OR ("glycemic control"):ti,ab,kw (Nurse led clinic):ti,ab,kw AND ("diabetes"):ti,ab,kw OR ("glycaemic control"):ti,ab,kw ("intervention studies"):ti,ab,kw AND ("standard care"):ti,ab,kw OR ("nurse's aide"):ti,ab,kw AND ("glycemic control"):ti,ab,kw
